# Dual functional construct containing kartogenin releasing microtissues and curcumin for cartilage regeneration

**DOI:** 10.1186/s13287-020-01797-2

**Published:** 2020-07-16

**Authors:** Negin Asgari, Fatemeh Bagheri, Mohamadreza Baghaban Eslaminejad, Mohammad Hossein Ghanian, Forogh Azam Sayahpour, Amir Mohammad Ghafari

**Affiliations:** 1 Department of Biomedical Engineering, Faculty of Chemical Engineering, Tarbiat Modares University, Tehran, Iran; 2 Department of Biotechnology, Faculty of Chemical Engineering, Tarbiat Modares University, Jalal Ale Ahmad Street, P.O.Box: 14115-111, Tehran, Iran; 3 Department of Stem Cells and Developmental Biology, Cell Science Research Center, Royan Institute for Stem Cell Biology and Technology, ACECR, Banihashem Sq., Banihashem St., Resalat Highway, P.O. Box 16635-148, Tehran, Iran; 4 Department of Cell Engineering, Cell Science Research Center, Royan Institute for Stem Cell Biology and Technology, ACECR, Tehran, Iran; 5 Center for Functional Materials, Faculty of Science and Engineering, Åbo Akademi University, Turku, Finland

**Keywords:** Cell aggregates, Microparticles, Kartogenin, Curcumin, Cartilage tissue engineering

## Abstract

**Background:**

Regeneration of articular cartilage poses a tremendous challenge due to its limited self-repair capability and inflammation at the damaged site. To generate the desired structures that mimic the structure of native tissue, microtissues with repeated functional units such as cell aggregates have been developed. Multicellular aggregates of mesenchymal stem cells (MSCs) can be used as microscale building blocks of cartilage due to their potential for cell-cell contact, cell proliferation, and differentiation.

**Methods:**

Chondrogenic microtissues were developed through incorporation of kartogenin-releasing poly (lactic-co-glycolic acid) (PLGA) microparticles (KGN-MP) within the MSC aggregates. The chondrogenic potential of KGN-MP treated MSC aggregates was proven in vitro by studying the chondrogenic markers at the RNA level and histological analysis. In order to address the inflammatory responses at the defect site, the microtissues were delivered in vivo via an injectable, anti-inflammatory hydrogel that contained gelatin methacryloyl (GelMA) loaded with curcumin (Cur).

**Results:**

The KGN-MPs were fabricated to support MSCs during cartilage differentiation. According to real-time RT-PCR analysis, the presence of KGN in the aggregates led to the expression of cartilage markers by the MSCs. Both toluidine blue (TB) and safranin O (SO) staining demonstrated homogeneous glycosaminoglycan production throughout the KGN-MP incorporated MSC aggregates. The curcumin treatment efficiently reduced the expressions of hypertrophy markers by MSCs in vitro. The in vivo results showed that implantation of chondrogenic microtissues (KGN-MP incorporated MSC aggregates) using the curcumin loaded GelMA hydrogel resulted in cartilage tissue regeneration that had characteristic features close to the natural hyaline cartilage according to observational and histological results.

**Conclusions:**

The use of this novel construct that contained chondrogenic cell blocks and curcumin is highly desired for cartilage regeneration.

**Graphical abstract:**

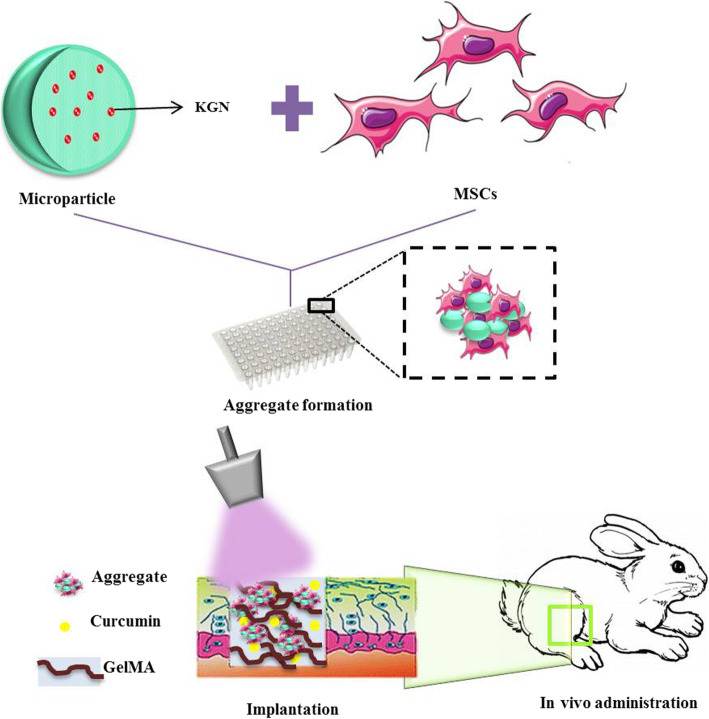

## Background

Articular cartilage lesions, particularly osteoarthritis (OA), affect millions of people worldwide. Cartilage is vulnerable to damage because it is an aneural and avascular tissue that has a slow rate of turnover and relatively low cellularity. There are few therapies for OA treatment and these include pharmaceutical management in addition to surgical treatments like micro-fracture, autografts as autologous chondrocyte implantation (ACI), and joint arthroplasty. Despite the improvements in designing suitable therapeutic strategies, these treatments could not restore the normal functions of the joints and are often not cost-effective [1, 2]. Tissue engineering is a promising way to improve the cartilage function or replace the damaged tissues. Traditional tissue engineering, as a top-down approach, almost has created a non-uniform cell distribution within a scaffold with a limitation in the cell and growth factor (GF) penetration. A bottom-up tissue engineering approach has been suggested to rebuild high-cell-density and efficient three-dimensional tissues. In this approach, a tissue is created from a gathering of microtissues or cellular building blocks [3, 4]. The aggregation of MSCs into the three-dimensional spheroids augmented their anti-inflammatory properties and has the potential for chondrocyte differentiation [5, 6]. Despite the advantages of cell aggregates, both limited diffusion of nutrients and GFs decrease both cell viability and differentiation, especially in the center of the structure. Several methods have been devised to circumvent the limited diffusion of GFs by incorporating GF-releasing polymer microparticles (MPs) or GF-encoded gene bounded MPs within the cell aggregates [7–10].

Small molecules are considered to be alternatives for GFs due to their simple structure, cost-effectiveness, and long half-life [11]. Kartogenin (KGN) is a small molecule that has been introduced for the induction of chondrocyte differentiation of mesenchymal stem cells (MSCs) by regulating the CBFβ-RUNX1 transcriptional program [12]. In the present study, we developed biodegradable polymer MPs for sustained release of KGN within MSC aggregates. These chondrogenic microtissues can be transplanted in vivo with minimally invasive strategies such as injection by a syringe needle.

On the other hand, the release of high levels of pro-inflammatory cytokines, such as tumor necrosis factor-α (TNF-α) and interleukin-1β (IL-1β), in the joint causes the manifestation of many OA symptoms and their circumvention is essential for successful regeneration [13]. Curcumin is a natural yellow-orange component derived from the rhizome of *Curcuma longa*. Researchers have proven the antioxidant, antitumor, and anti-inflammatory properties of curcumin [14–16]. Curcumin can block the action of nuclear factor-κB (NF-κB) [17] and prevent chondrocyte apoptosis through inhibition of caspase-3 [18]. NF-κB triggers the proteins involved in extracellular matrix degradation, inflammation, and apoptosis of chondrocytes. In this research, curcumin, an anti-inflammatory and anti-hypertrophy factor, was used to modulate the destructive microenvironment of the cartilage defect, which was assumed to be harmful for the viability of the transplanted cells. Hence, the curcumin was loaded in the hydrogel to provide a permissive microenvironment for the proper function of the chondrogenic MSCs.

KGN was encapsulated into the cell-sized, biodegradable poly (lactic-co-glycolic acid) (PLGA) MPs. The chondrogenic microtissues were prepared through incorporation of the KGN-releasing MPs within the MSC aggregates. The chondrogenic potential of KGN-MP treated MSC aggregates was confirmed in vitro. Next, the microtissues were transplanted in vivo through in situ encapsulation within a gelatin methacryloyl (GelMA) hydrogel that acted as a scaffold for microtissue engraftment and an inflammation modulator by localized delivery of curcumin. The results showed efficient cartilage regeneration in the joint in a skeletally mature rabbit model 12 weeks after implantation. Thus, the MP-engineered MSC aggregates along with curcumin could be utilized as the building blocks for a bottom-top tissue engineering approach in the regeneration of cartilage or other similar tissue defects.

## Materials and methods

### Preparation of kartogenin (KGN)-loaded poly (lactic-co-glycolic acid) (PLGA) microparticles (MPs)

The MPs were prepared by the oil-in-water (O/W) emulsion-solvent evaporation procedure. Concisely, 30 mg of PLGA (Resomer® RG504H, molecular weight 38–54 kDa, Sigma-Aldrich) was dissolved in 2.5 mL of dichloromethane and vortexed. Next, 3 mg of KGN (Tocris Bioscience) in dimethyl sulfoxide (DMSO) was added to the PLGA solution.

The solution was emulsified with 5 mL of 1% (w/v) polyvinyl alcohol (PVA; 87–90% hydrolyzed, average molecular weight 30–70 kDa, Sigma-Aldrich) and one drop of Tween 20. This emulsion was homogenized at 5000 rpm and subsequently added to the aqueous phase that included 100 mL of 0.1% (w/v) PVA, and stirred at 300 rpm at 37 °C for 3.5 h to evaporate the organic solvent. The MP suspension was washed in distilled water and harvested by centrifugation at 9000 rpm for 20 min at 4 °C. Supernatants were collected to calculate the encapsulation efficiency of KNG according to the following formula:

$$ \mathrm{Encapsulation}\ \mathrm{efficiency}\ \left(\%\right)=\left[\mathrm{actual}\ \mathrm{drug}\ \mathrm{loading}/\mathrm{total}\ \mathrm{drug}\ \mathrm{added}\right]\times 100 $$

The KGN-free MPs were prepared based on the same procedure. The particles were freeze-dried and stored at 4 °C for subsequent experiments. To increase the cell attachment, PLGA MPs were coated with gelatin through dispersion in a gelatin solution (0.1 mg/mL, Type B, Sigma-Aldrich) for 3 h, after which they were collected by centrifugation prior to cell culture.

### Characterization of kartogenin (KGN)-loaded poly (lactic-co-glycolic acid) (PLGA) microparticles (MPs)

The surface morphology and the size of the MPs were evaluated by scanning electron microscopy (SEM). The MPs were spread on a metal holder, gold-coated using an ion-sputtering device, and observed with a SEM (Phenom-World BV, 5652 AM Eindhoven, Netherlands). The mean diameter of the MPs (*n* = 100) was calculated using ImageJ (NIH image analysis software).

### In vitro drug release profile

The lyophilized KGN-MPs were dispersed in 2 mL of phosphate-buffered saline (PBS) solution and each tube contained 5 mg of particles. The release study was performed at 37 °C in an incubator.

At each sampling time, 1 mL of PBS was collected by centrifugation and the same volume of fresh PBS was replaced to examine the release kinetics of KGN from the MPs. The supernatants were stored at − 20 °C until analyzed. The concentration of released KGN was determined by UV spectrophotometry (Thermo Scientific, Multiskan Spectrum, 51118650) at 278.4 nm based on a standard curve.

### Stem cell aggregate cultures

#### Rabbit mesenchymal stem cell (MSC) isolation and characterization

Rabbit bone marrow was obtained from 3- to 4-month-old New Zealand white rabbits according to a procedure approved by the Animal Care and Use Committee of Royan Institute, Tehran, Iran. First, the animals were anesthetized by intramuscular injections of 1.5 mL ketamine (100 mg/mL) and 0.5 mL xylazine (20 mg/mL). Bone marrow was aspirated under aseptic conditions from the tibia medullary canal by using a 19-gauge needle. Then, the bone marrow was mixed with Dulbecco’s modified Eagle’s medium (DMEM; Gibco) that contained 1% penicillin and streptomycin (PAN-Biotech) and 15% fetal bovine serum (FBS; Gibco) and incubated at 37 °C and 5% CO_2._ The medium was changed twice a week until cells reached confluency. Passage-3 cells were used for the experiments.

To assess the osteogenic and adipogenic capability of the isolated cells, the medium culture was substituted by osteogenic medium (50 μg/mL ascorbic acid 2-phosphate [Sigma-Aldrich], 10 nM dexamethasone [Sigma-Aldrich], and 10 mM β-glycerol phosphate [Sigma-Aldrich]) and adipogenic medium (100 nM dexamethasone, 50 μg/mL ascorbic acid 2-phosphate, and 50 μg/mL indomethacin [Sigma-Aldrich]), respectively, for 21 days. Then, the cells were fixed and stained with Alizarin Red and Oil Red O (Sigma, USA) and observed by light microscopy.

#### Preparation of mesenchymal stem cell (MSC) aggregates and microparticle (MP)-incorporated MSC aggregates

In order to prepare the non-adherent culture plates, we coated the plate surfaces with PVA. One wt% PVA in PBS was added to the 96-well PCR plates (100 μL/well) and the plates were incubated at 37 °C for 15 min. Then, the PVA solution was aspirated and the coated wells were washed with PBS prior to culturing the cells on the plates.

MP (3 × 10^4^ particles) and MSC (3 × 10^4^ cells) suspensions were separately prepared in standard medium and added to the wells. For MSC aggregates, the cells were added to each well without the MPs. The plates were incubated in 5% CO_2_ at 37 °C, and the medium was replaced every other day.

#### Microparticle (MP) incorporation efficiency

The incorporation efficiency of the MPs was measured by counting the particles in each well before and after aggregate formation with a hemocytometer. To observe the spatial distribution of particles within the cell aggregates, Rhodamine B (Sigma-Aldrich)-labeled MPs were utilized to produce the cell aggregates. The aggregates were fixed in 4% paraformaldehyde and cut into 10-μm thick sections using a cryostat (Leica CM 1850). The cells were stained with 4′,6-diamidino-2-phenylindole (DAPI, Abcam) dye for 1 min and washed twice in PBS before imaging.

#### Morphological assessment of the aggregates

The cellular aggregates were primarily observed under a bright-field optical microscope (IX71 Olympus) and the size of the aggregates was measured at various time points during the culture period by ImageJ software.

Cellular organization and spheroid structure also were assessed by SEM. The aggregates were fixed in 2.5% of glutaraldehyde for 24 h, dehydrated in a series of graded ethanol dilutions, and thoroughly dried and imaged.

#### Live/dead assay

MP cytotoxicity was assessed by the live/dead assay. After 14 days of culture, the aggregates were stained with a solution that contained 1 μM calcein AM and 2 μM ethidium homodimer (Invitrogen) at room temperature for 30 min. Then, the solution was removed, and the aggregates were washed with PBS and imaged using a fluorescent microscope (IX71 Olympus).

#### MTT assay

We used 3-(4,5-dimethylthiazol-2-yl)-2,5-dimethyltetrazolium bromide (MTT, Sigma-Aldrich) to measure cell viability and the proliferation rate of the cells based on cell metabolic activity during 1, 7, 14, and 21 days of the cell culture. For the MTT assay, each aggregate was incubated for 2 h in medium that contained 0.5 mg/mL of MTT. Viable cells could reduce the MTT to a formazan pigment. These purple formazan crystals were dissolved in 100 μl of DMSO and the absorbance was read using a UV/Vis microplate reader at 570 nm.

### In vitro chondrogenic differentiation analyses

#### Real-time RT-PCR

Total RNA was prepared from the aggregates on days 14 and 21 of differentiation using TRIzol reagent (Invitrogen). To obtain sufficient RNA concentrations for real-time RT-PCR analysis, 30 aggregates were pooled for each test. Complementary DNA (cDNA) was synthesized by reverse transcription of 1 μg of total RNA using a cDNA kit (Takara, P3-T7) according to the manufacturer’s instructions. A total of 25 ng of cDNA was amplified using specific primers and the SYBR Green Master Mix with a real-time PCR system (Applied Biosystems, ABI). Three independent biological replicates were analyzed for each sample. The expression level of each gene was calculated using the 2^−(ΔΔCT)^ method with glyceraldehyde 3-phosphate dehydrogenase (*GAPDH*) as the reference gene. MP-free aggregates were used as the control.

Real-time RT-PCR was used to assess the effects of curcumin on hypertrophy and cartilage marker gene expressions in the MSC aggregates. We also examined the expressions of the hypertrophic cartilage remodeling factors *MMP13* and *MMP1* after curcumin treatment.

Supplementary Table S1 lists the primers used to amplify each gene of interest.

#### Histological analyses

After 14 days of culture, the aggregates were encapsulated in 2% agar, fixed in 4% paraformaldehyde, and processed and embedded in paraffin. Five-micrometer sections of the aggregates were mounted on the slides. The slides were deparaffinized and dehydrated and subsequently stained with hematoxylin and eosin (H&E), toluidine blue (TB), or safranin O (SO). Hematoxylin stains the cell nuclei a blue color whereas eosin stains the extracellular matrix and cytoplasm a pink color. Both SO and TB are cationic dyes that bind to sulfated glycosaminoglycans (sGAGs) [19]. The stained sections were observed under a light microscope (Olympus, Japan).

#### Sulfated glycosaminoglycan (sGAG) quantification

The total contents of sGAGs secreted during chondrogenic differentiation of MSCs were determined quantitatively using 1,9-dimethylmethylene blue (DMMB; Sulfated Glycosaminoglycan Assay Kit, Blyscan™). The GAG content in the samples was calculated against a standard curve supplied by the kit.

After 14 days, the aggregates were digested overnight with papain in a sodium phosphate buffer that contained 0.2 M Na_2_HPO_4_- NaH_2_PO_4_, 0.05 M EDTA, and cysteine-HCl (5 mM) at pH 6.4 and 60 °C. Then, the dye solution was added to 100 μl of the papain-digested solution. After 30 min, the sample was centrifuged to deposit the sGAG-dye complex. The dissociation reagent was added and the absorbance was measured at 656 nm by an ELISA reader (Thermo Scientific, Multiskan Spectrum, 51118650).

### In vivo study

#### In vivo osteochondral defect model

All of the animal procedures were approved by the Animal Care and Use Committee of Royan Institute, Tehran, Iran. The rabbits were first anesthetized by intramuscular injections of 1.5 mL ketamine (100 mg/mL) and 0.5 mL xylazine (20 mg/mL). Full-thickness cartilage defects (5 mm in diameter, 5 mm in depth) were created in the centers of the trochlear grooves using a micromotor in both knees of the mature male New Zealand white rabbits (weight, 2.0–2.5 kg). The osteochondral defects involve both cartilage and adjacent underlying bone. These defects receive bone marrow-derived MSCs for repair after injury, but mechanically, inferior fibrocartilage fills the defect. The full-thickness cartilage defect size has been defined as 3 mm in rabbit; however, there are some reports of spontaneous healing. Consequently, we created large full-thickness osteochondral defects that were 5 mm in diameter and 5 mm in depth [20].

Cell aggregates in the different groups were encapsulated in GelMA and injected into the defect site. GelMA was synthesized and polymerized according to protocols published in the literature [21, 22]. First, gelatin was dissolved at 10% (w/v) in Dulbecco’s phosphate-buffered saline (DPBS; Gibco) at 55 °C. A high degree of methacrylation was accomplished by the addition of 20% (w/v) Methacrylic anhydride (MA) to the mixture. MA was added slowly (0.5 mL/min) and the mixture was stirred for 3 h. The mixture was dialyzed against distilled water using dialysis tubing for 1 week at 40 °C to remove the salts and any unreacted MA. Finally, the solution was freeze-dried for 2 days and stored at − 80 °C.

The rabbits’ knees were divided into four groups: sham (treated only by GelMA hydrogel), [MSC] Agg (MSC aggregates encapsulated in a GelMA hydrogel), [MSC/KGN-MP] Agg (KGN-MP incorporated MSC aggregates encapsulated in a GelMA hydrogel), and Cur + [MSC/KGN-MP] Agg (KGN-MP incorporated MSC aggregates encapsulated in a curcumin-loaded GelMA hydrogel). The concentration of curcumin was 20 μM (Sigma Aldrich) in the GelMA hydrogels in the last group, which we selected based on our MTT results.

After injection of the hydrogel precursor (10% GelMA solution in PBS) and photoinitiator (1-[4-(2-hydroxyethoxy)-phenyl]-2-hydroxy-2-methyl-1-propanone; Irgacure 2959) into the defect sites, we then exposed these defects to UV irradiation (350 nm) at a 10 w/cm^2^ intensity for 5 min and then sutured the defect. The animals were returned to their cages and allowed to move freely. The limbs were permitted to fully weight bear. The rabbits were sacrificed at 1 and 3 months for macroscopic and histological evaluations of the treated knees.

#### Gross morphology assessment

The knees were separated and imaged for quantitative evaluation by the International Cartilage Repair Society (ICRS) gross morphology assessment score [23]. The gross appearance of each knee was evaluated in terms of the degree of defect repair or filling, integration same as the surrounding cartilage, and macroscopic appearance (rough or smooth surface) by two independent observers who were blinded to the group assignments. Each item was scored between 0 to 4 points with respect to the degree of repair for a total score of 12 points.

#### Histology evaluation

The separated knees were fixed in 10% formalin, decalcified in 4% EDTA, and embedded in paraffin. Next, 5-μm sections were obtained from the center of each defect, and these sections were stained with H&E, TB, and Masson’s trichrome. The sections were evaluated under a light microscope (Olympus, Japan).

### Statistical analysis

All of the data were expressed as mean ± standard deviation from at least three biological replicates. Differences between any two groups were determined by one-way ANOVA in PRISM software. *P* values less than 0.05 were considered to be statistically significant.

## Results

### Fabrication and characterization of the KGN-loaded poly (lactic-co-glycolic acid) (PLGA) microparticles (MPs)

**Fig. 1:**
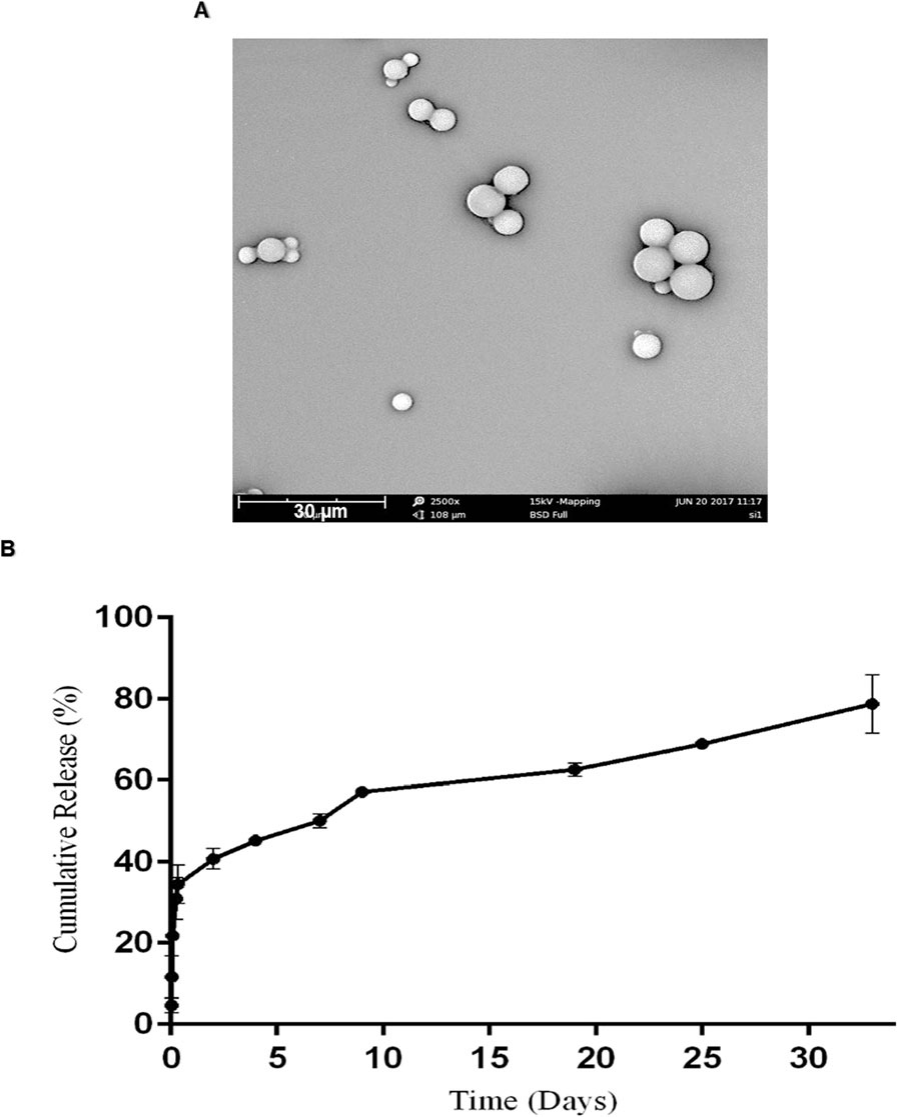
Microparticle (MP) characterization. **a** Scanning electron microscopy (SEM) images of MPs show their smooth surface morphologies. The mean diameter of the particles was 11 ± 5.5 μm. **b** Kartogenin **(**KGN) release profile from MPs for 32 days. Data are expressed as mean ± standard deviation (*n* = 3)

The KGN-loaded PLGA MPs were fabricated by a single emulsion/solvent evaporation method. SEM was used to characterize the morphology and to determine the size of the PLGA MPs (Fig. 1a). SEM images of the MPs showed that they had a regular and smooth spherical morphology. According to the SEM images, the particles had an average diameter of 11 ± 5.5 μm, which was in the range of the previously reported MPs for incorporation within cell spheroids [24, 25].

Analysis of KGN loading revealed that the drug was effectively encapsulated into the MPs with an encapsulation efficiency of 70%, which was expected due to the hydrophobic nature of KGN. The release profile of KGN from KGN-loaded MPs was followed for 32 days (Fig. 1b). A burst release was observed during the first day followed by a sustained trend over a 32-day period. The release mechanism might be a combination of drug diffusion and polymer degradation.

### Incorporation of microparticles (MPs) within the mesenchymal stem cell (MSC) aggregates

**Fig. 2:**
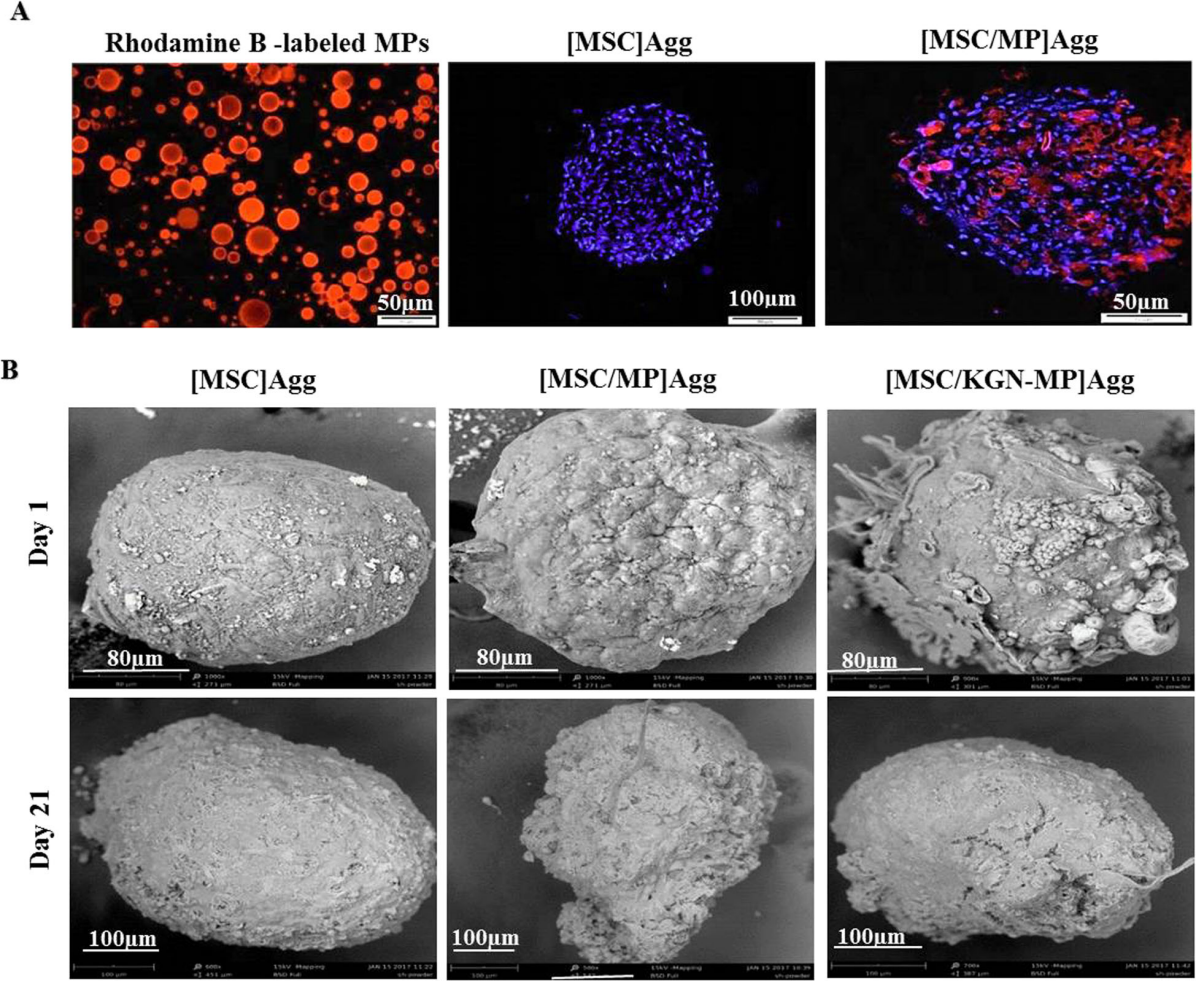
Preparation and characterization of mesenchymal stem cell (MSC) aggregates and MSC/microparticle (MP) co-aggregates. **a** Left to right: Rhodamine B-labeled MPs; fluorescent microscopic image of a cell aggregate stained with 4′,6-diamidino-2-phenylindole (DAPI); and distribution of MPs within the cell aggregates. **b** Scanning electron microscopy (SEM) images of the cell aggregates on days 1 and 21

The MP-incorporated MSC aggregates were prepared by simple mixing of the gelatin-coated MPs and single MSCs during cell aggregate formation. An efficient incorporation and aggregate formation was obtained at an approximately 1:1 cell to MP ratio. The incorporation efficiency measurement showed that more than 75% of the MPs were incorporated within the MSC aggregates after 48 h. Analysis of the fluorescent signal from sections of the aggregates showed homogenous distribution of Rhodamine B-labeled MPs throughout the MSC aggregates ([MSC/MP] Agg group) (Fig. 2a). The incorporated MPs can release up to approximately 100 nM of KGN during the differentiation culture period (14 days) based on the release curve and the encapsulation efficiency.

Figure S1 shows light microscopy images of the MSC aggregates and [MSC/MP] co-aggregates with or without KGN after 1 and 21 days of incubation in non-attachable PVA coated 96-well plates. The average size of the aggregate increased from 200 ± 21 μm to 300 ± 32 μm after 21 days due to cell proliferation or ECM secretion. The surface morphology of the MSC aggregates, with or without MPs, was evaluated by SEM (Fig. 2b). A coarse surface was observed in the presence of MPs in the aggregates on the first day and this was not detected after 21 days.

### Viability and proliferation of cells in the aggregate culture

**Fig. 3:**
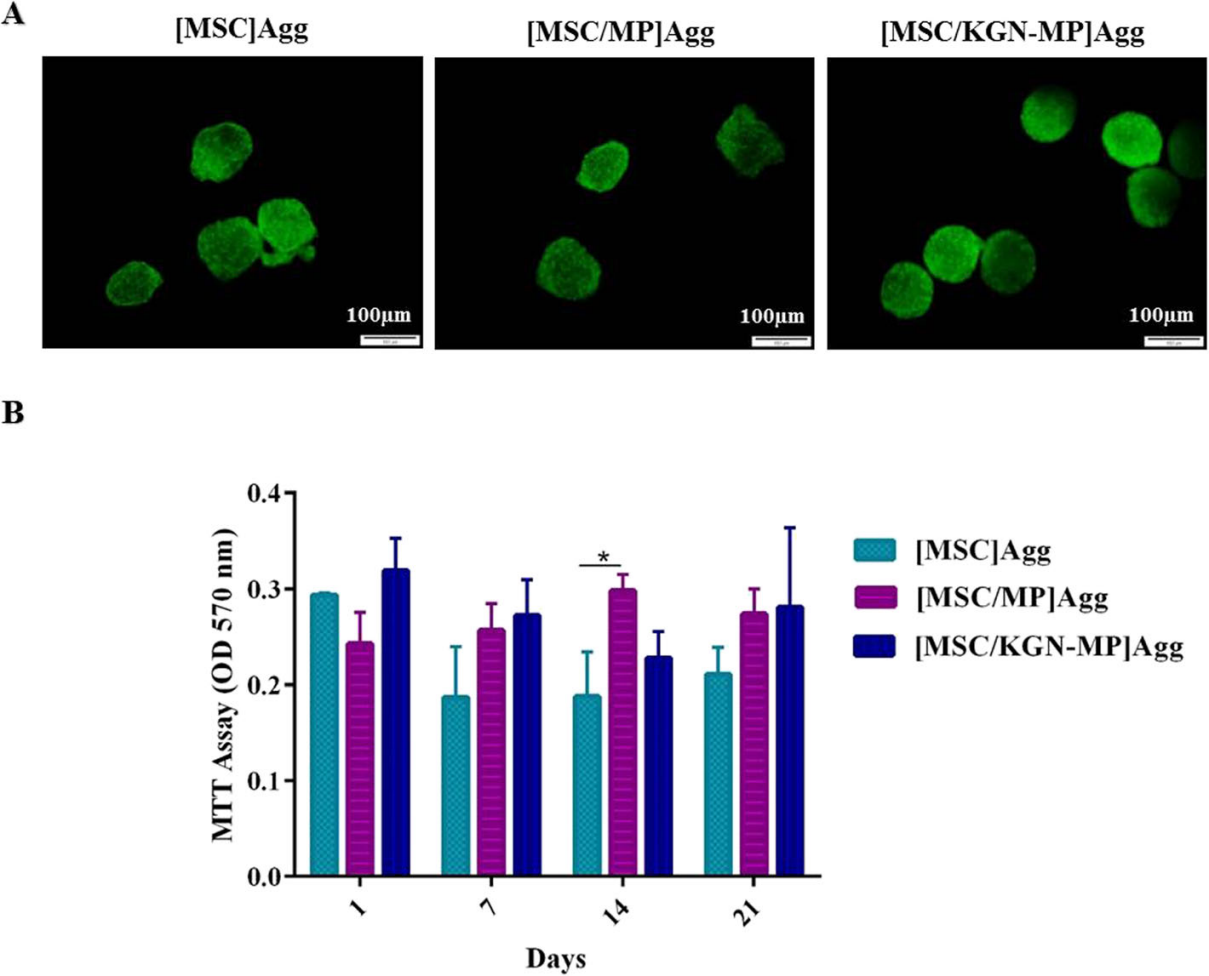
Viability and metabolic activity of the cell aggregates. **a** Live/dead assay using calcein AM and ethidium homodimer stains in the aggregate cultures (green: live, red: dead) **b** Viability in aggregates was determined by the MTT assay at days 1, 7, 14, and 21.

The effect of MP incorporation on MSC viability was investigated by the live/dead assay (Fig. 3a). After 14 days, we observed few dead cells in the experimental groups proposing the lack of cytotoxic effect of MPs and KGN in aggregates. Also, other than day 14, there was no significant difference in the cell viability results according to the MTT assay between the cell aggregate group and aggregates in the presence of MPs (Fig. 3b). The MPs seem to be capable of working as spacers in the cell aggregates, which facilitate the penetration of oxygen and nutrients into the cell aggregates.

### In vitro chondrogenic differentiation analyses

**Fig. 4:**
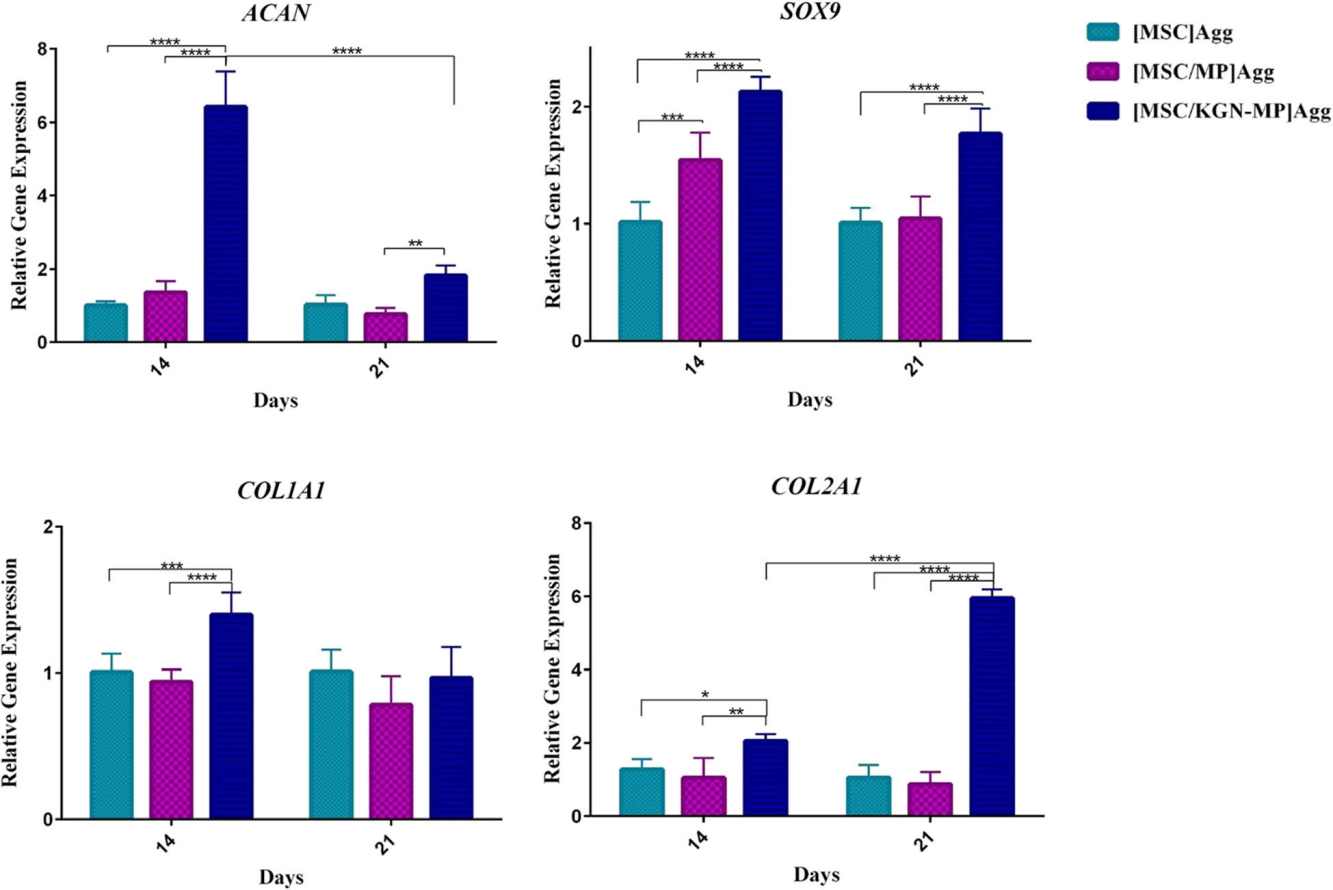
Gene expression in the mesenchymal stem cell (MSC) aggregate cultures. The relative expressions of *ACAN*, *COL1A1*, *COL2A1*, and *SOX9* by real-time RT-PCR on days 14 and 21 after aggregation. mRNA levels were normalized to glyceraldehyde 3-phosphate dehydrogenase (*GAPDH*) levels and are presented as fold changes compared to MSC aggregates alone. Data are shown as mean ± SD (*n* = 3). *p < 0.05, **p < 0.01, ***p < 0.001, ****p < 0.0001

We sought to evaluate the effect of KGN releasing MPs in cell aggregates on the chondrogenic differentiation of MSCs. Expressions of the chondrocyte markers collagen type Π (*COL2A1*), SRY-box 9 (*SOX 9*), and Aggrecan (*ACAN*) were analyzed by real-time RT-PCR after 14 and 21 days of culture. The presence of KGN in the aggregates led to expression of the cartilage markers by MSCs, which suggested an efficient effect of KGN in stimulating the cartilage-forming capacity of the MSCs (Fig. 4). The expression of *COL1A1*, as a fibrocartilage marker, was not high in the presence of KGN; however, in the chondrogenic medium that contained transforming growth factor beta 1 (TGF-β1), the expression of this gene increased 11-fold on day 14 and 31-fold on day 21 compared to the control (cell aggregate only) (Fig. S2).

**Fig. 5:**
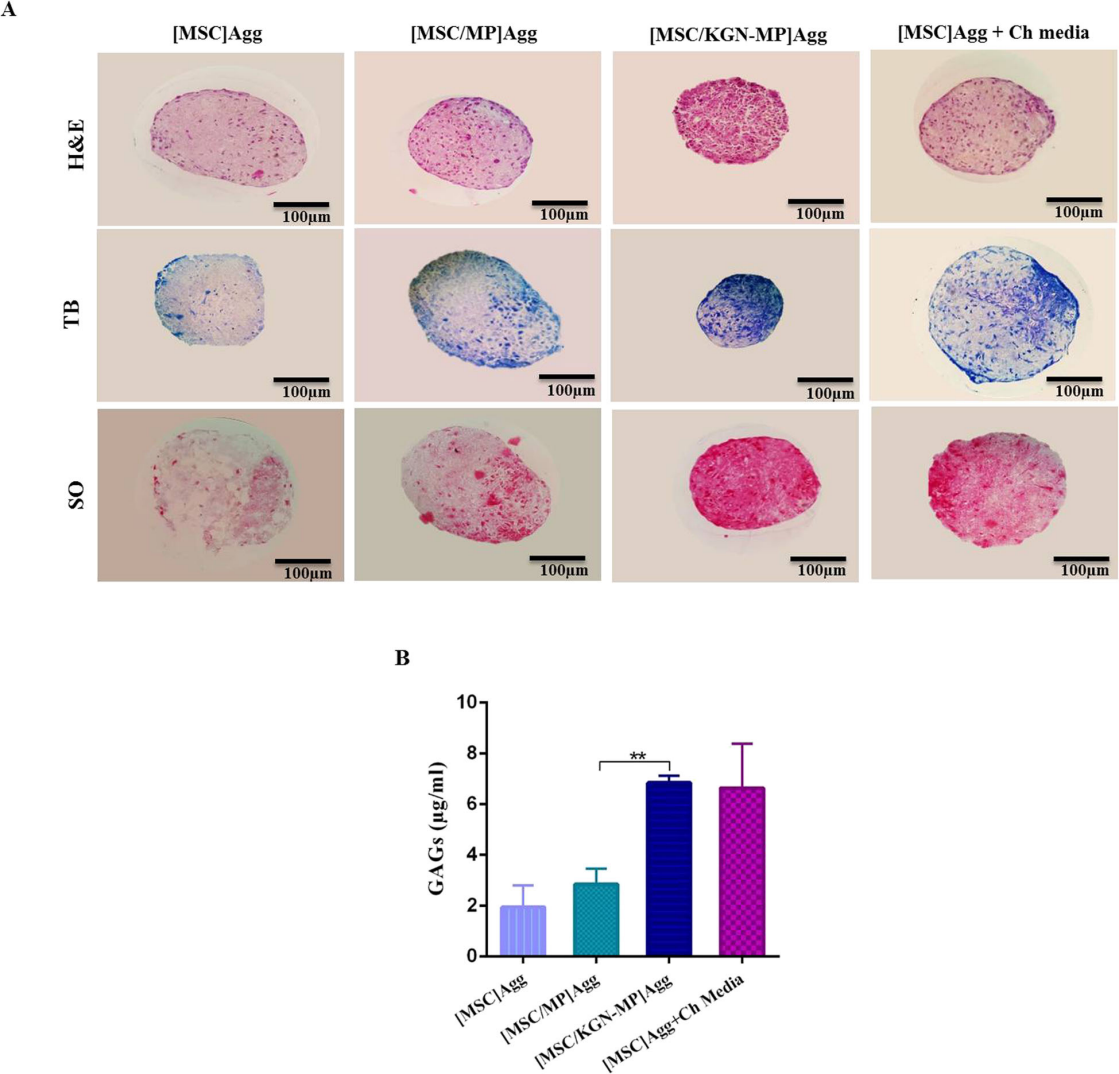
Cartilage matrix production in the presence of KGN-loaded microparticles (MPs) in mesenchymal stem cell (MSC) aggregates. **a** Histological evidence of chondrogenesis in the [MSC] Agg, [MSC/MP] Agg, [MSC/KGN-MP] Agg and [MSC] Agg + Ch media groups after 14 days. **b** Sulfated glycosaminoglycan (sGAG) content in the aggregate groups. Data are presented as mean ± SD, (*n* ≥ 3). **p < 0.01

Histological analysis showed that the [MSC] Agg group cultured in cartilage medium and [MSC/KGN-MP] Agg group were positive for SO and TB staining after 2 weeks of culture. In the MSC/KGN loaded MPs cultures, proteoglycan distribution appeared more homogeneously throughout the co-aggregate cultures compared to the MSC aggregates cultured in the cartilage medium where positive staining was mostly observed in the outer layer (Fig. 5a).

Figure 5b shows the GAG amount in the different groups on day 14. Release of KGN from the MPs enhanced sGAG production in the MSC aggregates. No significant differences in sGAG content in the MSC aggregates and KGN-free MP/MSC aggregates demonstrated that the blank MPs did not influence the chondrogenic differentiation of the MSCs.

### The effect of curcumin on chondrogenic differentiation and hypertrophy of mesenchymal stem cells (MSCs)

According to the literature, the nontoxic concentration of curcumin varies depending on the cell type [17]. The effect of different concentrations of curcumin (10–40 μM) on the viability of rabbit bone marrow MSCs was measured by the MTT assay after 48 h of incubation at 37 °C. We observed that curcumin showed no significant cytotoxicity up to 20 μM; therefore, this concentration was considered safe and used for the additional experiments. More than 90% of the cells were alive after treatment with 20 μM of curcumin (Fig. S3).

**Fig. 6:**
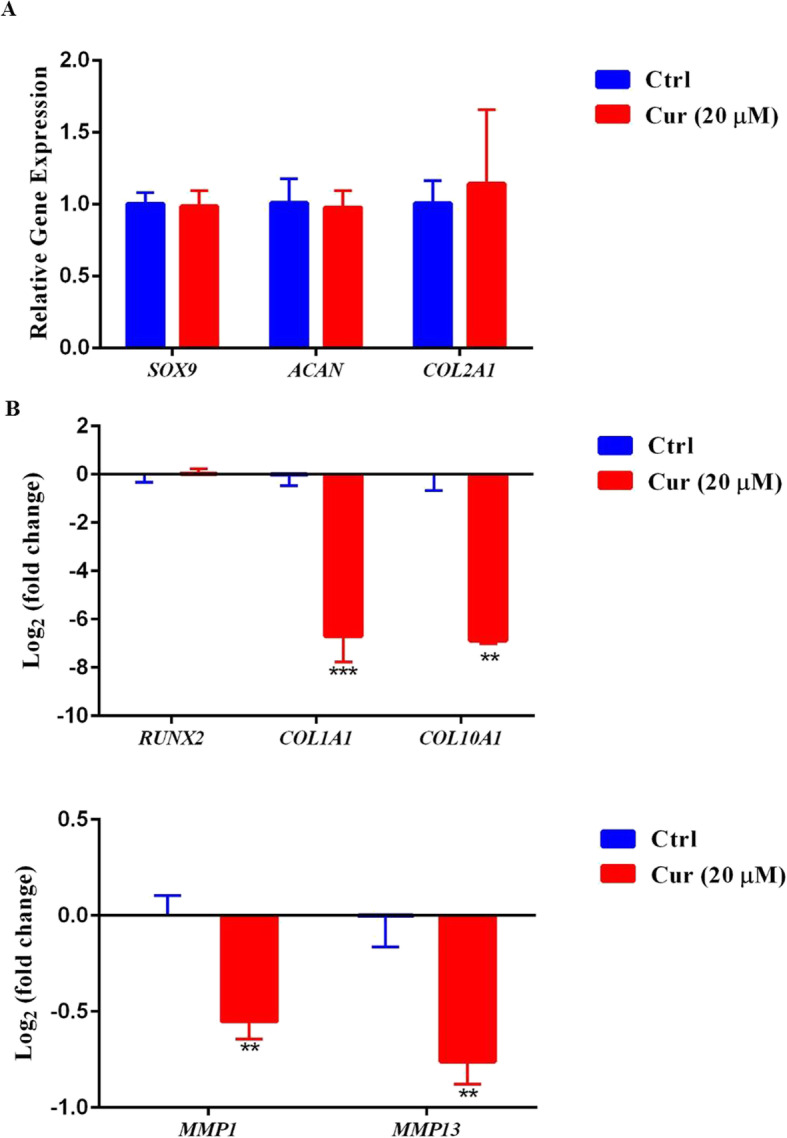
Gene expressions in the mesenchymal stem cell (MSC) aggregates in the presence of curcumin. **a** Relative expressions of chondrogenic markers by real-time RT-PCR 14 days after treatment. **b** Relative expression of hypertrophy genes in log 2 scale. The mRNA levels were normalized to glyceraldehyde 3-phosphate dehydrogenase (*GAPDH*) levels and presented as fold changes compared to untreated MSCs

The effect of curcumin on chondrogenic differentiation of the MSC aggregates was evaluated by real-time RT-PCR on day 14 (Fig. 6a). The results showed that curcumin had no effect on the differentiation of MSCs to chondrocytes at this concentration. The expression levels of *COL2A1*, *SOX 9*, and *ACAN* did not change after administration of curcumin.

The expression of hypertrophic and matrix devastating genes *COL10A1*, *MMP13*, and *MMP1* were alleviated in the presence of curcumin. *COL1A1*, a fibrocartilage marker, was also reduced after curcumin treatment (Fig. 6b).

### In vivo findings

**Fig. 7:**
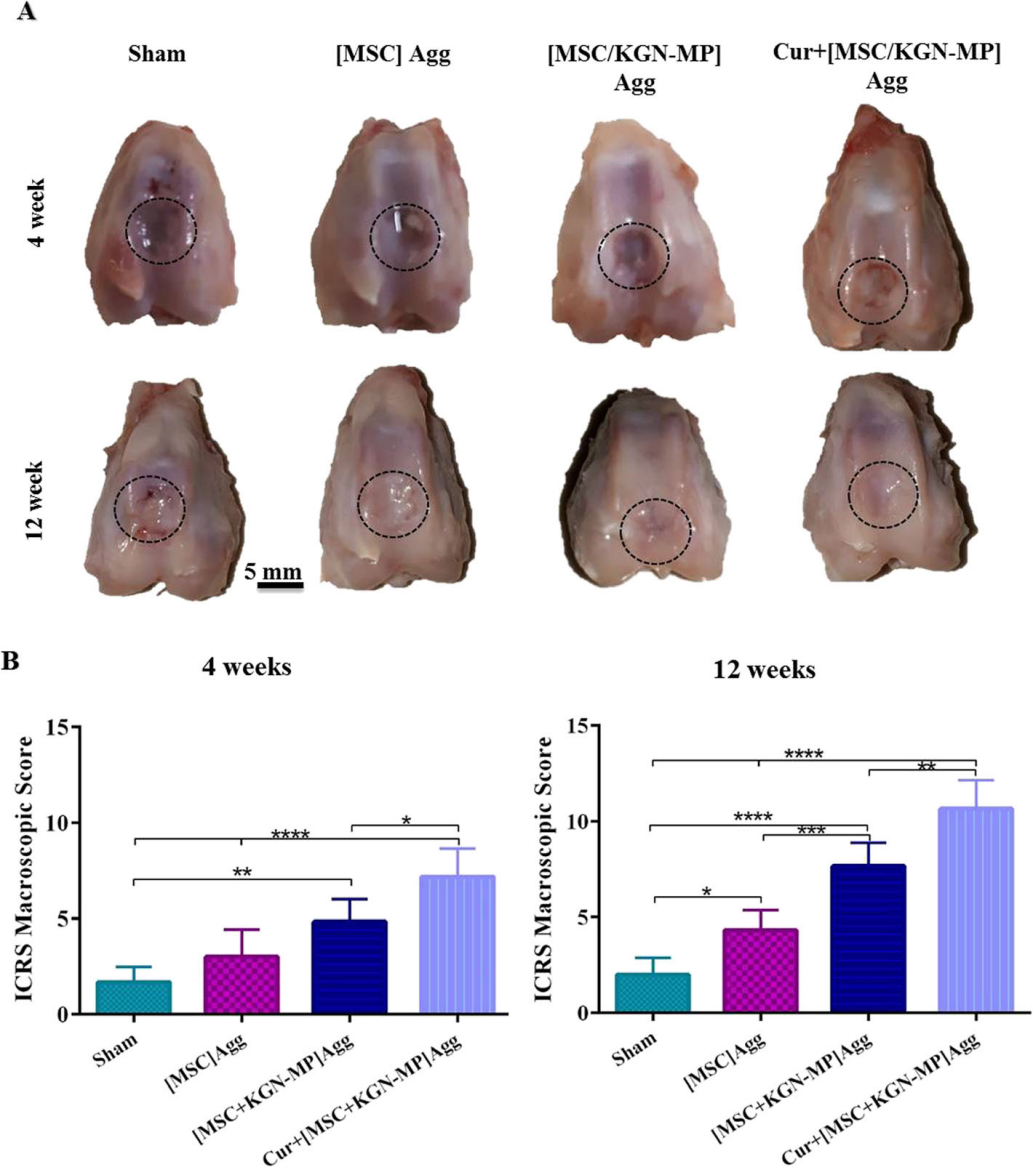
Macroscopic assessments of the repaired defect. **a** Photographs of repaired cartilage at 4 and 12 weeks after implantation. The dash lines indicate the defect site. **b** International Cartilage Repair Society (ICRS) macroscopic scores of the repaired cartilages at 4 and 12 weeks. Data are shown as mean ± SD (*n* = 3). *p < 0.05; **p < 0.01; ***p < 0.001

The rabbit model was used to evaluate the effectiveness of this novel construct in repairing cartilage defects. No rejection, infection, or extensive fibrosis was observed in any of the experimental groups. At 12 weeks post-implantation, the cartilage surface was relatively smooth and bright in the groups treated with [MSC + KGN – MP] Agg and Cur + [MSC/KGN-MP] Agg compared to the sham group. In these groups, the defect areas were fully filled with hyaline cartilage-like tissue, which was similar to the neighboring native cartilage without any gaps, whereas some cleavages were observed in the sham group (Fig. 7a). This result was confirmed by the ICRS macroscopic scores that were calculated to evaluate the repaired cartilage (Fig. 7b). The Cur + [MSC/KGN-MP] Agg group had the significantly highest ICRS macroscopic scores at the evaluated times.

**Fig. 8:**
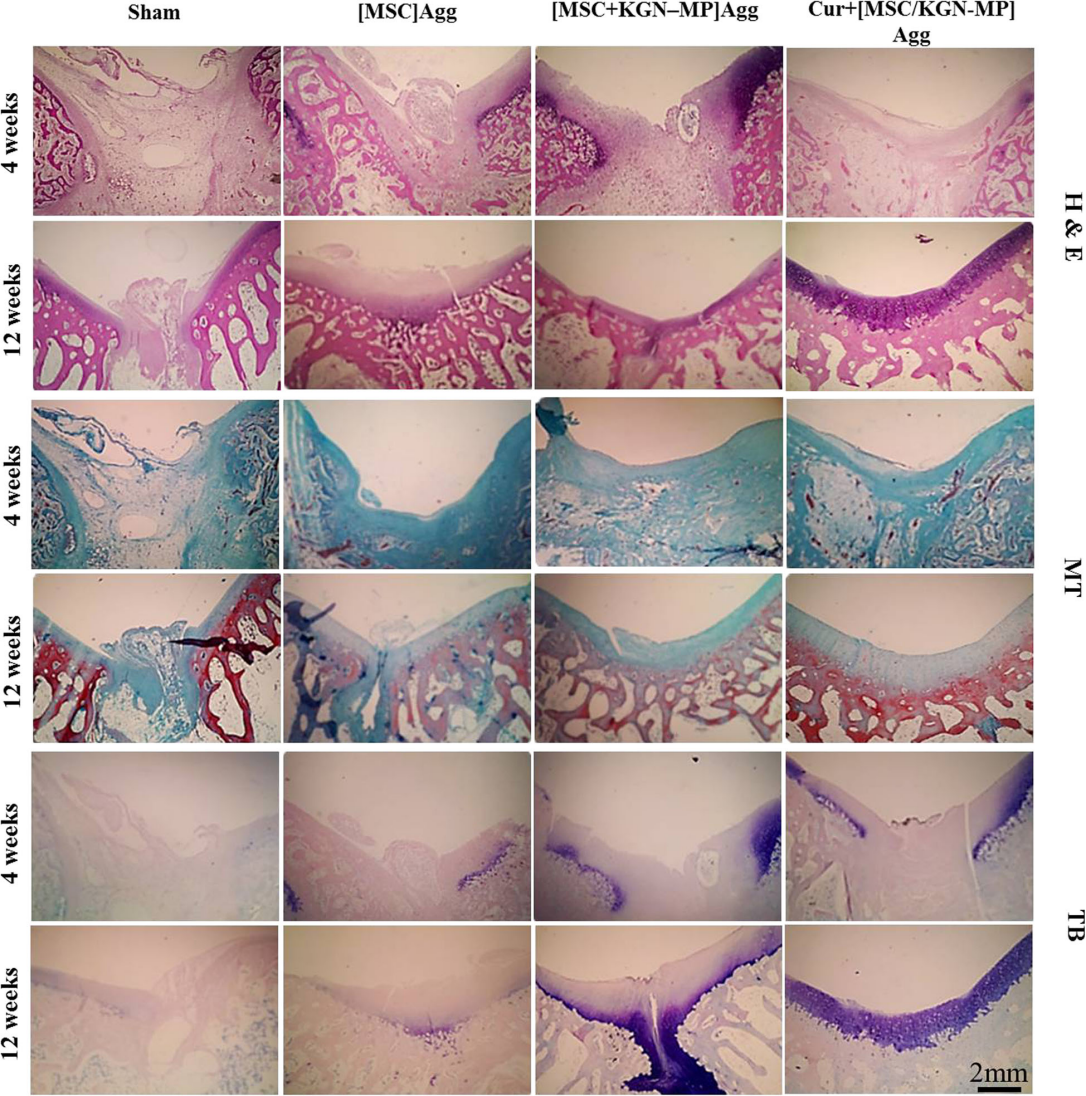
Histological findings of rabbit osteochondral defect repair at 4 and 12 weeks after treatment. Hematoxylin and eosin (H&E), Masson’s trichrome (MT), and toluidine blue (TB) staining at 4 and 12 weeks after implantation

Histological results showed that in the Cur + [MSC/KGN-MP] Agg group, the defect was completely replaced by hyaline-like cartilage at 12 weeks after implantation. This finding was not observed in the other groups. In this group, the articular surface was smooth at 12 weeks post-implantation (Fig. 8). The cell arrangement in this group was similar to normal cartilage and the cells had lacunae. The repaired tissue in the sham group was irregular and abnormal compared to other groups. Weak TB staining was observed for glycosaminoglycans in the [MSC] Agg and sham groups 4 and 12 weeks post-implantation, whereas the Cur + [MSC/KGN-MP] Agg group showed strong staining 12 weeks after implantation (Fig. 8).

## Discussion

It is well-documented that “top-down” tissue engineering approaches have some limitations in the preparation of homogeneous and intricate microstructural tissues [4]. To overcome these limitations, it would be more appropriate to use structures from smaller components with associated cells and inducing factors [26]. For chondrogenic differentiation of MSCs, the aggregation culture is a preferable choice because it mimics the compact cell-cell interactions [27, 28]. These cell aggregates can be used as building blocks of a “bottom-up” approach for creating a functional tissue. The results of several studies have shown that incorporation of MP in cell aggregates could increase nutrient permeability. MPs, as a scaffold, mimic the micro-environment of the cell and provide an active surface for cell attachment. Eventually, they can be used as a carrier for controlled release of differentiation factors [25, 29–34].

Several reports describe the application of nano/MPs (NP/MP) in KGN delivery as carriers. Kang et al. covalently conjugated KGN to chitosan for its intra-articular delivery. Their results suggested that chitosan-KGN NPs effectively induced chondrogenic differentiation of human bone marrow MSCs [35]. Shi et al. used hyaluronic acid scaffold integrated with KGN-loaded PLGA NPs in a cell-free approach to treat a cartilage defect [36]. Su et al. employed PLGA microspheres for the controlled release of KGN in a collagen-based scaffold. The cultured cells on the scaffolds showed good viability and chondrogenic differentiation capacity based on in vitro results [37]. Recently, Almeida et al. evaluated the effects of surface chemistry on NP characteristics in terms of hydrophobicity, size, and surface charge. Three NP formulations (PLGA, PLGA-PEG, and PLGA-PEG-HA) were fabricated and their effects on differentiation were assessed on monolayer cultures of MSCs in culture plates. Despite the dissimilarity between NP properties, there was no significant difference between the treated groups in terms of sGAG production [38].

In the current study, KGN-loaded MPs efficiently integrated within the forming MSC aggregates to play a double role as drug carriers and spacers for nutrient diffusion. The presence of KGN-loaded MPs inside the cell aggregates resulted in uniform chondrogenic differentiation. To the best of our knowledge, this is the first report that has described the integration of cell-sized MPs within cell aggregates for delivery of KGN. This strategy can be used to simultaneously deliver cells and inducing factors to the defect site.

The results of this study revealed that the KGN-loaded MPs could be used as an efficient and cost-effective approach for direct differentiation of MSC aggregates into chondrocytes. Real-time RT-PCR results showed that collagen type II and aggrecan, as the most essential components of the ECM in native articular cartilage, were highly upregulated in the MSC aggregates treated with KGN-MPs. For eligible articular tissue engineering, it is important to avoid the fibrocartilage phenotype during differentiation. Our results showed that chondrogenic differentiation in the MSC aggregates treated with KGN-MPs was not accompanied by *COL1A1* expression, whereas in routine chondrogenic media supplemented with TGF-β1, there was an elevated expression of *COL1A1*, as a marker of fibrocartilage. The role of TGF-β1 on MSC osteogenic differentiation was assessed by Zao et al. Their results showed that treatment with TGF- β1 increased the levels of the osteoblast differentiation markers. At the molecular level, the crucial regulators of bone differentiation and formation are RUNX2 and transcriptional coactivator with PDZ-binding motif (TAZ). TGF-β1 stimulated TAZ expression at both the mRNA and protein levels [39]. TGF-β signaling also has a role in osteoblast differentiation and bone formation during development [40]. Based on this evidence, KGN might possibly be more specific in terms of cartilage differentiation of MSCs and could be a proper alternative to chondrogenic growth factors. Recently, Kwon et al. reported that KGN treatment could induce IL-10 production and showed protective activity against cartilage degeneration through downregulation of matrix metalloproteinases (MMPs), MMP3 and MMP6, and inflammatory agents such as IL-6, TNF-α, and IL-1β [41].

Our histological results and quantification of GAG content showed a significantly higher proteoglycan deposition in the KGN-MPs loaded aggregates compared to the other groups, which confirmed the gene expression findings. These results were comparable with a study by Ravindran et al. who reported the differentiation of stem cell spheroids through MP-mediated delivery of TGF-β3 [8]. TGF-β is a protein with a very short half-life that loses its functions both in vitro and in vivo within hours; however, the continuous presence of this protein is necessary for differentiation of MSCs to chondrocytes. Small molecules can be a suitable alternative for GFs; thus, in the present research, the small-molecule KGN was selected as a differentiation factor. Recently, Li et al. showed that KGN-incorporated thermogels were an appropriate microenvironment for effective cartilage regeneration in a rabbit model [42].

Our results demonstrated the homogenous distribution of GAG in KGN-MP treated MSC aggregates compared to MSC aggregates treated with common chondrogenic media. In the classic treatment of MSC aggregates in culture medium, the inductive factors are delivered to aggregates by an outside-in approach. As a result, the cells in the peripheral layers have better accessibility to nutrients and differential factors. However, in the center of the aggregates, the cells depend on the diffusion process for the exchange of nutrients and GFs, which functions only for a limited distance. Conversely, an inside-out delivery approach, through MP-mediated release of inductive factors (herein, small molecules) directly within the cell aggregates, can circumvent the mass transport limitation.

These chondrogenic cell blocks were implanted to the defect site in the rabbit knee via an injectable hydrogel supplemented with curcumin. In our study, for the first time, the simultaneous effect of KGN and curcumin was assessed in repairing the cartilage defect in vivo.

We employed GelMA as the scaffold for implantation and maintenance of MSC aggregates and localized delivery of curcumin in the defect site. The microenvironment of damaged cartilage is poisoned with inflammatory and hypertrophy factors. Pro-inflammatory cytokines, such as TNF-α and IL-1β, force the chondrocytes to secrete MMPs. These enzymes are the main actors in degradation of collagen type II and aggrecans in the cartilage extracellular matrix as well as cytokines, GFs, and receptors. Under this condition, chondrocytes switch to a hypertrophic phenotype that triggers the endochondral ossification process. Thus, the cell therapy approach is not effective because there is no guarantee for the viability and maintenance of the cells at the defect site. To this end, it is essential to overcome the inflammatory and hypertrophic situation for the treatment of cartilage defects [43, 44]. Curcumin is a highly pleiotropic molecule with powerful antioxidant, anticancer, and anti-inflammatory properties. Its ability for the treatment of numerous inflammatory diseases such as OA and rheumatoid arthritis (RA) has been reported [45, 46]. Buhrmann et al. showed that curcumin impedes the adverse effects of proinflammatory cytokines in OA disease through suppression of NF-κB. They demonstrated that curcumin inhibited the inflammatory situation and reduced apoptosis in MSCs; therefore, it could create a suitable microenvironment for chondrogenic differentiation of progenitor cells and MSCs in vivo [17]. Csaki et al. investigated the effects of curcumin with or without resveratrol on viability and expression of chondrogenic genes in the primary human chondrocytes in vitro. They showed that curcumin abolished IL-1β-induced apoptosis and inflammation. They also demonstrated that both resveratrol and/or curcumin prevented downregulation of *COLII* and *SOX9* in an inflammatory situation that contained IL-1β [47]. Recently, Kim et al. reported that curcumin downregulated the expressions of the inflammatory factors COX-2, TNF-α, and IL1-β in rabbit chondrocytes [48]. Some reports have also indicated that MSCs tend to obtain a hypertrophic phenotype during chondrogenic induction [49]. The expression of hypertrophy markers during cartilage differentiation is a major challenge in MSC-based cartilage regeneration because hypertrophy can be followed by vascular attack and bone formation [50]. Our results depicted that curcumin down-regulated the hypertrophic genes including *COL10A1* and *MMP 13*.

Our in vivo results in the osteochondral defect in the rabbit model demonstrated that KGN-MP loaded MSC-aggregates considerably facilitated cartilage regeneration. The best regeneration was observed in the group that received both chondrogenic cell aggregates and curcumin. The Cur + [MSC/KGN-MP] Agg group also exhibited successful integration into the host cartilage, whereas no efficient tissue regeneration was observed in the sham group after 12 weeks.

## Conclusion

Here we showed the development of chondrogenic microtissues. These microtissues were formed by aggregates that contained a combination of MSCs and KGN loaded MPs, which could be transplanted in vivo by minimally invasive strategies. The use of KGN-loaded MP/MSC aggregates increased the production of components of the cartilage ECM. Effective regeneration was observed by the simultaneous delivery of chondrogenic cell aggregates and curcumin in the osteochondral defect model. Curcumin could modulate the hypertrophic and inflammatory environment of the defect site. In conclusion, this combination might provide the desired treatment for cartilage tissue engineering applications.

## Supplementary information

**Additional file 1: Table S1.** Primer sequences used for real time RT-PCR. **Figure S1.** Light microscopic images of cell aggregates on days 1 and 21. **Figure S2.***COL1A1* and *COL2A1* expression in MSCs aggregate cultures. The expression of *COL1A1* and *COL2A1* in [MSC/KGN-MP] Agg group and [MSC] Agg in the chondrogenic medium compared to the control (cell aggregate). The ratio of *COL2/COL1* expression showed the effects of KGN in hyaline cartilage formation. **Figure S3.** Cell viability of MSCs at various concentrations of curcumin (0, 10, 20, 30 and 40 μM) was examined by MTT assay for 48 h. Data are shown as mean ± SD, **p < 0.01; ****p < 0.0001.

## Data Availability

All data generated or analyzed during this study are included in this published article and its supplementary information files.

## Abbreviations

PLGA: Poly (lactic-co-glycolic acid)
MSCs: Mesenchymal stem cells
OA: Osteoarthritis
ACI: Autologous chondrocyte implantation
TNF-α: Tumor necrosis factor-α
IL-1β: Interleukin-1β
GelMA: Gelatin methacryloyl
SEM: Scanning electron microscopy
PBS: Phosphate-buffered saline
DMEM: Dulbecco’s modified Eagle’s medium
PVA: Poly (vinyl alcohol)
DAPI: 4′,6-diamidino-2-phenylindole
MTT: 3-(4,5-dimethylthiazol-2-yl)-2,5-dimethyltetrazolium bromide
DMSO: Dimethyl sulfoxide
cDNA: Complementary DNA
GAPDH: Glyceraldehyde 3-phosphate dehydrogenase
H&E: Hematoxylin and eosin
TB: Toluidine blue
SO: Safranin O
sGAGs: Sulfated glycosaminoglycans
DMMB: 1,9-dimethylmethylene blue
DPBS: Dulbecco’s phosphate-buffered saline
ICRS: International Cartilage Repair Society
TGF-β1: Transforming growth factor beta 1
TAZ: Transcriptional coactivator with PDZ-binding motif
