# Supplementary material for: Dual functional construct containing kartogenin releasing microtissues and curcumin for cartilage regeneration
Source: Stem Cell Res Ther. 2020 Jul 16;11:289. doi: 10.1186/s13287-020-01797-2 (PMC7367357; doi:10.1186/s13287-020-01797-2)
Supplement: Supplementary file 1 — Additional file 1: Table S1. Primer sequences used for real time RT-PCR. Figure S1. Light microscopic images of cell aggregates on days 1 and 21. Figure S2.COL1A1 and COL2A1 expression in MSCs aggregate cultures. The expression of COL1A1 and COL2A1 in [MSC/KGN-MP] Agg group and [MSC] Agg in the chondrogenic medium compared to the control (cell aggregate). The ratio of COL2/COL1 expression showed the effects of KGN in hyaline cartilage formation. Figure S3. Cell viability of MSCs at various concentrations of curcumin (0, 10, 20, 30 and 40 μM) was examined by MTT assay for 48 h. Data are shown as mean ± SD, **p < 0.01; ****p < 0.0001. [file 13287_2020_1797_MOESM1_ESM.docx]

Supplementary data

**Dual Functional Construct Containing Kartogenin Releasing Microtissues and Curcumin for Cartilage Regeneration**

Negin Asgari^a^, Fatemeh Bagheri^b^*, Mohammadreza Baghaban Eslaminejad^c^*, Mohammad Hossein Ghanian^d^, Forogh Azam Sayahpour^c^, Amir Mohammad Ghafari^d,e^

a. Department of Biomedical Engineering, Faculty of Chemical Engineering, Tarbiat Modares University, Tehran, Iran

b. Department of Biotechnology, Faculty of Chemical Engineering, Tarbiat Modares University, Tehran, Iran

c. Department of Stem Cells and Developmental Biology, Cell Science Research Center, Royan Institute for Stem Cell Biology and Technology, ACECR, Tehran, Iran.

d. Department of Cell Engineering, Cell Science Research Center, Royan Institute for Stem Cell Biology and Technology, ACECR, Tehran, Iran

e. Center for Functional Materials, Faculty of Science and Engineering, Åbo Akademi University, Turku, Finland

**Table S1) Primer sequences used for real time RT-PCR.**

| **Name of gene** | **Primer Sequence** |
| --- | --- |
| ***ACAN*** | FOR: 5ˈ GGAGGTCGTGGTGAAAGGTG 3ˈ  Rev: 5ˈ CTCACCCTCCATCTCCTCTG 3ˈ |
| ***COL2A1*** | FOR:5' GTGGAAGAGCGGTGACTAC 3'  REV:5' TAGGTGATGTTCTGGGAGC 3' |
| ***SOX9*** | FOR: 5ˈ AAGATGACCGACGAGCAG 3ˈ  REV: 5ˈ GGCTTGTTCTTGCTGGAG 3ˈ |
| ***COL1A1*** | FOR:5ˈGGTGCTGCTGGTAAAGAAGG 3ˈ  REV: 5ˈGTCTACCCAAAGCACCAG 3ˈ |
| ***RUNX2*** | FOR: 5ˈ GGACTGTGGTTACTGTCATGG ‎3'  REV: 5ˈ GTGAAACTCTTGCCTCGTCC ‎3' |
| ***COL10A1*** | FOR: 5ˈAGTTCTTCATTCCCTATGCCA3ˈ  REV: 5ˈCAATGTCTCCTTTCGGTCCA3ˈ |
| ***MMP1*** | FOR: 5ˈ TTCCAAAGCAGAGAGGCAAT 3'  REV: 5ˈ GCTTCCCAGTCACTTTCAGC 3' |
| ***MMP13*** | FOR:5ˈTTGGAACTAAAGAACATGGCGA3ˈ  REV:5ˈCGGTGTAGGTGTAGATGGGA3ˈ |
| ***GAPDH*** | FOR:5ˈCACCCACTCCTCTACCTTCG3'  REV:5ˈGGTCTGGGATGGAAACTGTG3' |


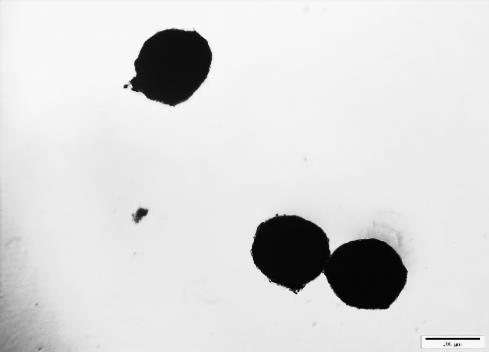

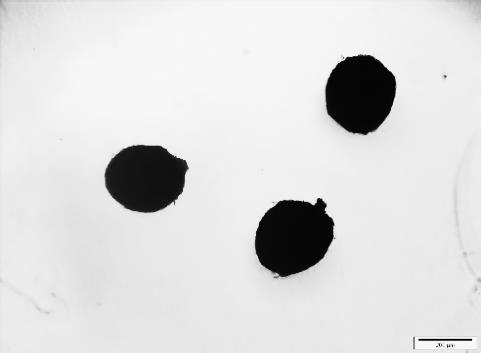

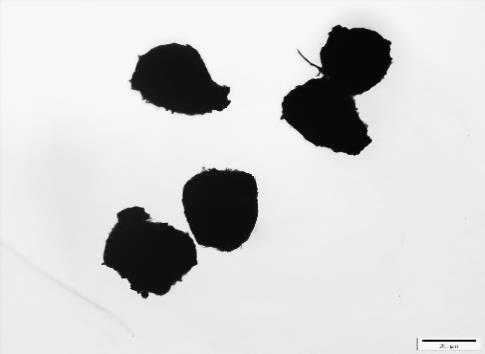

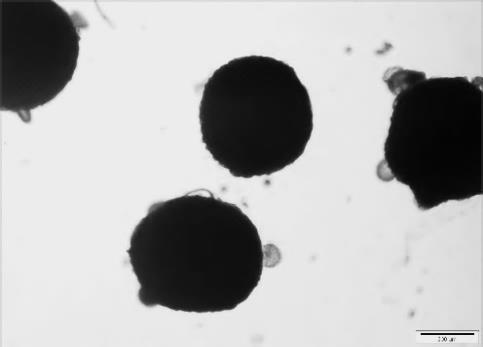

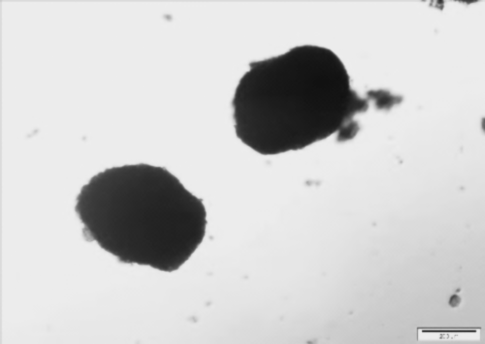

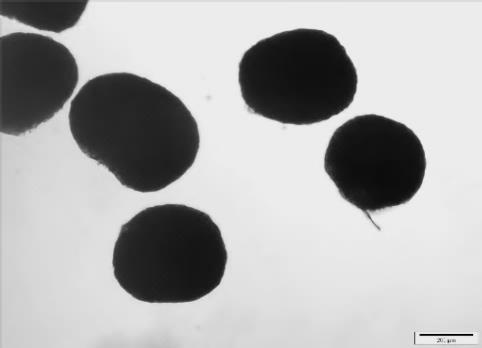


**Day 1**

**Day 21**

**[MSC]Agg**

**[MSC/MP]Agg**

**[MSC/KGN-MP]Agg**

**200 µm**

**200 µm**

**200 µm**

**200 µm**

**200 µm**

**200 µm**

**Fig S1)** Light microscopic images of cell aggregates on days 1 and 21


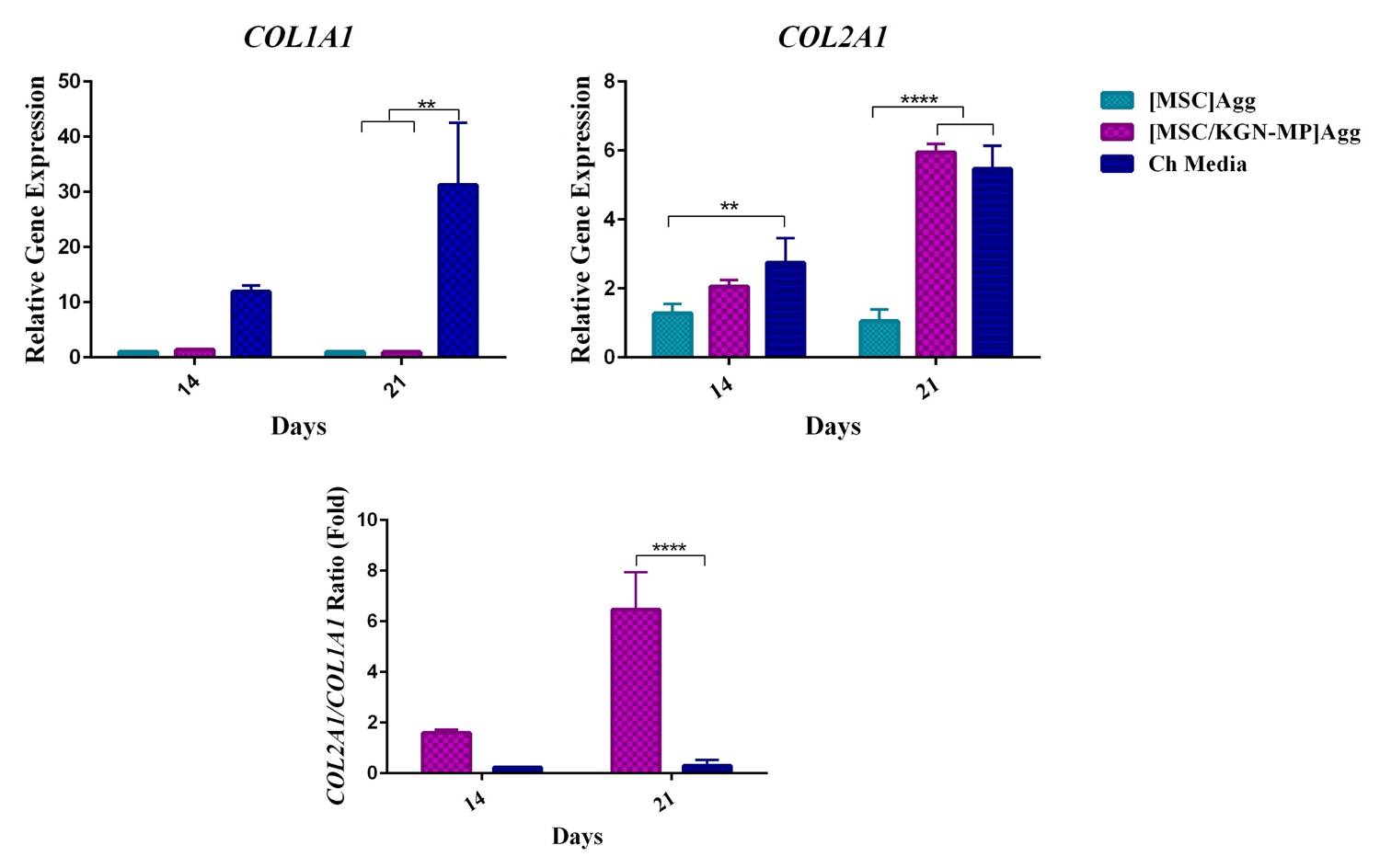


**Fig S2)** *COL1A1* and *COL2A1* expression in MSCs aggregate cultures. The expression of *COL1A1* and *COL2A1* in [MSC/KGN-MP]Agg group and [MSC]Agg in the chondrogenic medium compared to the control (cell aggregate). The ratio of *COL2/COL1* expression showed the effects of KGN in hyaline cartilage formation.


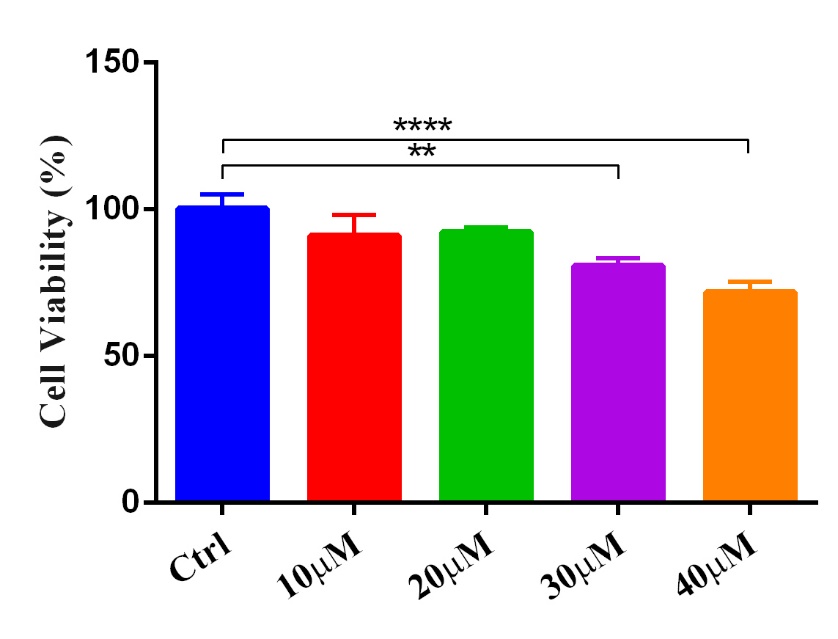


**Fig S3)** Cell viability of MSCs at various concentrations of curcumin (0, 10, 20, 30 and 40 μM) was examined by MTT assay for 48 h. Data are shown as mean ± SD, **p < 0.01; ****p < 0.0001
